# Evolutionary Comparison of the Complete Chloroplast Genomes in *Convallaria* Species and Phylogenetic Study of Asparagaceae

**DOI:** 10.3390/genes13101724

**Published:** 2022-09-26

**Authors:** Qi-Xiang Lu, Xiao Chang, Jing Gao, Xue Wu, Jing Wu, Zhe-Chen Qi, Rui-Hong Wang, Xiao-Ling Yan, Pan Li

**Affiliations:** 1 Zhejiang Province Key Laboratory of Plant Secondary Metabolism and Regulation, College of Life Sciences and Medicine, Zhejiang Sci-Tech University, Hangzhou 310018, China; 2 The Key Laboratory of Conservation Biology for Endangered Wildlife of the Ministry of Education, and Laboratory of Systematic & Evolutionary Botany and Biodiversity, College of Life Sciences, Zhejiang University, Hangzhou 310058, China; 3 Shaoxing Academy of Biomedicine, Zhejiang Sci-Tech University, Shaoxing 312366, China; 4 Eastern China Conservation Centre for Wild Endangered Plant Resources, Shanghai Chenshan Botanical Garden, Shanghai 201602, China

**Keywords:** *Convallaria*, chloroplast genome, comparative analysis, phylogenomics, Asparagaceae

## Abstract

The genus *Convallaria* (Asparagaceae) comprises three herbaceous perennial species that are widely distributed in the understory of temperate deciduous forests in the Northern Hemisphere. Although *Convallaria* species have high medicinal and horticultural values, studies related to the phylogenetic analysis of this genus are few. In the present study, we assembled and reported five complete chloroplast (cp) sequences of three *Convallaria* species (two of *C. keiskei* Miq., two of *C. majalis* L., and one of *C. montana* Raf.) using Illumina paired-end sequencing data. The cp genomes were highly similar in overall size (161,365–162,972 bp), and all consisted of a pair of inverted repeats (IR) regions (29,140–29,486 bp) separated by a large single-copy (LSC) (85,183–85,521 bp) and a small single-copy (SSC) region (17,877–18,502 bp). Each cp genome contained the same 113 unique genes, including 78 protein-coding genes, 30 transfer RNA genes, and 4 ribosomal RNA genes. Gene content, gene order, AT content and IR/SC boundary structure were nearly identical among all of the *Convallaria* cp genomes. However, their lengths varied due to contraction/expansion at the IR/LSC borders. Simple sequence repeat (SSR) analyses indicated that the richest SSRs are A/T mononucleotides. Three highly variable regions (*petA*-*psbJ*, *psbI*-*trnS* and *ccsA*-*ndhD*) were identified as valuable molecular markers. Phylogenetic analysis of the family Asparagaceae using 48 cp genome sequences supported the monophyly of *Convallaria*, which formed a sister clade to the genus *Rohdea*. Our study provides a robust phylogeny of the Asparagaceae family. The complete cp genome sequences will contribute to further studies in the molecular identification, genetic diversity, and phylogeny of *Convallaria*.

## 1. Introduction

The monocot genus *Convallaria* L. (Asparagaceae) are perennial herbs commonly found in the understory of temperate deciduous forests in the Northern Hemisphere [1,2]. Three morphologically similar but isolated species with different geographical distribution were recognized in the genus, namely *C. keiskei*, *C. majalis*, and *C. montana* [3,4]. *Convallaria keiskei* is widely distributed in Sakhalin, Korea, China, Japan and Eastern Siberia [5,6]. *Convallaria majalis*, commonly known as Lily of the Valley, is native to Europe and now widely ranged in the temperate regions of Eurasia and Eastern North America [7]. *C. montana*, the American Lily of the Valley, has a limited distribution endemic to the southern Appalachian Mountains in North America [5,6]. This genus is characterized by a 10–15 cm leaf length, two leaves and a raceme of about 10 white flowers on the stem apex, 4–10 mm bracts length, nearly globose seeds and a base chromosome number of 18 [8]. Like many perennial herbaceous plants, the *Convallaria* species reproduces asexually by rhizome and sexually by seed [9]. They always form extensive colonies by spreading underground stems [10,11]. The *Convallaria* species is widely cultivated as an ornamental plant for its white bell-shaped flowers and sweet fragrance [7]. However, potential poisonings are a concern in terms of the cardenolide glycosides found throughout these plants, such as convallatoxin and convallatoxol [12]. These components could have medicinal value. For example, steroidal glycosides extracted from *C. majalis* whole plants had the potential for use as an anti-lung cancer agent [13]. *Convallaria keiskei* plants was used in the treatment of salivary gland cancer as an efficient therapeutic alternative [14]. Although *Convallaria* species have high medicinal and horticultural values, studies on the phylogenetic relationships and evolution of these *Convallaria* species are few.

Asparagales is the largest order of monocots, though in APG III [15], the number of families recognized has fallen from 26 to 14 [16]. In previous research, the phylogeny of Asparagaceae was reconstructed using cp or nuclear loci, with low support value for the position of *Convallaria* [17,18]. In recent years, chloroplast genomes have been widely used in reconstructing the phylogenetic relationships among plant groups for their uniparental inheritance, lack of recombination, and conservativeness in gene content and gene order [19,20,21]. Many phylogenetic relationships that remained unresolved with few loci have been clarified by using whole cp genome sequences, especially in recently diverged plant groups [22,23,24,25]. Furthermore, the fast-evolving regions of the cp genome can be utilized as DNA barcodes to identify the morphological similar species, as well as molecular markers for systematic and phylogeographic analyses [26,27]. Thus, phylogenetic analyses with cp genomes offer an efficient method to obtain a robust phylogenetic relationship of the Asparagaceae for further evolutionary study.

In this study, we report and annotate five complete cp genomes of three *Convallaria* species. We aimed to (1) investigate the global structure patterns of *Convallaria* cp genomes; (2) analyze the repeat sequences and SSRs among the five cp genomes; (3) screen hotspot DNA regions; and (4) reconstruct the phylogenetic relationships within Asparagaceae to locate the phylogenetic position of *Convallaria* and confirm its monophyly.

## 2. Materials and Methods

### 2.1. Plant Samples, DNA Extraction, and Sequencing

Fresh leaves of five individual plants (two *C. keiskei*, two *C. majalis* and one *C. montana*) were collected in the field and dried with silica gel immediately. Voucher specimens were deposited in the Herbarium of Zhejiang Sci-Tech University. Total genomic DNA was extracted from ~10 mg leaf tissue using the CTAB extraction protocol with modification [28]. After purification, the extracted DNA was used to generate short-insert (<800 bp) paired-end sequencing libraries according to the Illumina standard protocol (Illumina, San Diego, CA, USA). The genomic DNA of each individual specimen was indexed by tags and pooled together in one lane of Hiseq 2500 (Illumina) for sequencing at Beijing Genomics Institute (BGI, Shenzhen, China). After sequencing and data treatment, 14,576,340–21,266,964 clean reads with pair-end 125 bp read length were retrieved for the five cp genomes.

### 2.2. Chloroplast Genome Assembly and Annotation

The cp genomes were assembled with both de novo and reference guided approaches [29]. The cp genome sequence of *Rohdea chinensis* (MH356725; [30]) obtained from NCBI was used as a reference (http://www.ncbi.nlm.nih.gov/) (accessed on 5 February 2020). Firstly, we assembled the clean reads into contigs on the de novo assembler using CLC Genomics Workbench with the following optimized parameters: bubble size of 98, minimum contig length of 200, mismatch cost of 2, deletion and insertion costs of 3, length fraction of 0.9, and similarity fraction of 0.8. Next, all the contigs were aligned to the reference genome using local BLAST (http://blast.ncbi.nlm.nih.gov/) (accessed on 12 February 2020) with ≥90% similarity and query coverage. The contigs of each individual were aligned with *R. chinensis* genome and ordered in Geneious v11.1.2 (http://www.geneious.com) (accessed on 15 February 2020) to construct the draft chloroplast genomes. Then, the clean reads were mapped to the draft genome sequences again to check the contigs’ concatenation. Finally, the complete chloroplast genome sequences were obtained by connecting these overlapping contigs. The yielded cp genomes were annotated through the online program Dual Organellar Genome Annotator (DOGMA; [31]. The annotated organelle genomes of *C. keiskei* (A4, A118), *C. majalis* (A63, A69 and *C. montana*(A114) were deposited in GenBank (accession numbers ON645923, ON303653, ON303655, ON645922 and ON303654). The start and stop codons and the exon–intron boundaries of the encoded genes were accurately confirmed by comparison with homologous genes of *R. chinensis* using MAFFT v7 [32]. In addition, the tRNAscan-SE v1.21 was used to verify the tRNA genes with default parameters [33]. The graphic maps of the cp genomes of *Convallaria* were drawn in Organellar Genome DRAW [34].

### 2.3. Comparative Analysis of Convallaria cp Genomes and Hotspot Regions Identification

We used the five complete chloroplast genomes of *Convallaria* for comparative analyses. MAFFT v7 was used in the alignment of the plastid genome sequences [32]. Boundary regions between the LSC, IR and SSC and their lengths were compared and analyzed using cp genomes. The sequence identity of the five *Convallaria* cp genomes was plotted using the online software mVISTA (http://genome.lbl.gov/vista/mvista/submit.shtml) (accessed on 22 February 2020) with the Shuffle-LAGAN mode [35]. The cp DNA rearrangement analysis of the five chloroplast genomes were conducted using Mauve Alignment [36]. In order to screen the fast-evolving regions among the cp genomes, the sequence alignment was subjected to a sliding window analysis to evaluate the total number of mutations (*Eta*) and nucleotide diversity (*Pi*) for all protein-coding and intergenic spacer regions using the DNA Polymorphism calculation in DNASP v5.1 [37]. Regions that met the following two criteria were used: (1) an aligned length > 200 bp; and 2) mutation site > 0. We also calculated the *Eta* and *Pi* among the Asparagaceae species to obtain the hypervariable regions which could be used in future molecular evolutionary and systematic studies of this family.

### 2.4. Long Repeats and Simple Sequence Repeats

The position and size of three repeat sequence types, including direct (forward), inverted (palindromic) and reverse repeats, were identified in the five cp genomes of *Convallaria* using the online program REPuter [38] (accessed on 10 March 2020). For the above repeat types, we set the constraints in REPuter as the following: (1) repeat size longer than 30 bp; and (2) sequence identity more than 80%, with a hamming distance of 3 (i.e., the gap size between repeats larger than 3 bp). SSRs were detected using MIcroSAtellite (MISA) perl script [39] with a threshold for mono-, di-, tri-, tetra-, penta-, and hexanucleotide SSRs containing 10, 5, 5, 3, 3, and 3 repeat units, respectively.

### 2.5. Phylogenetic Analysis

The 5 cp genomes of *Convallaria* and other 43 cp genomes of Asparagaceae downloaded from NCBI were recovered to infer their phylogenetic relationships within this family (Table S1). *Agapanthus coddii* was used as the outgroup. Multiple alignment of coding sequences from 48 cp genomes were performed using MAFFT v7 [32]. The phylogenetic reconstructions were applied using two methods: maximum likelihood (ML) and Bayesian inference (BI). The best-fitting models (GTR + F + R3) of nucleotide substitutions were determined by the Bayesian Information Criterion (BIC) yielded using jModelTest v2.1.7 [40]. The ML analyses were performed in RAxML-HPC v8.2.20 [41] with 5000 bootstrap (BS) replicates. Bayesian inference (BI) analyses were run with MrBayes v.3.2.5 [42]. The Markov chain Monte Carlo (MCMC) algorithm was run for 1,000,000 generations. Trees were sampled every 500 generations. The first 25% trees were discarded as burn-in. A consensus tree was obtained from the remaining trees, and we estimated the posterior probabilities (PPs) and visualized them in FigTree v1.4.2.5.

## 3. Results

### 3.1. Genome Organization and Features

**Figure 1:**
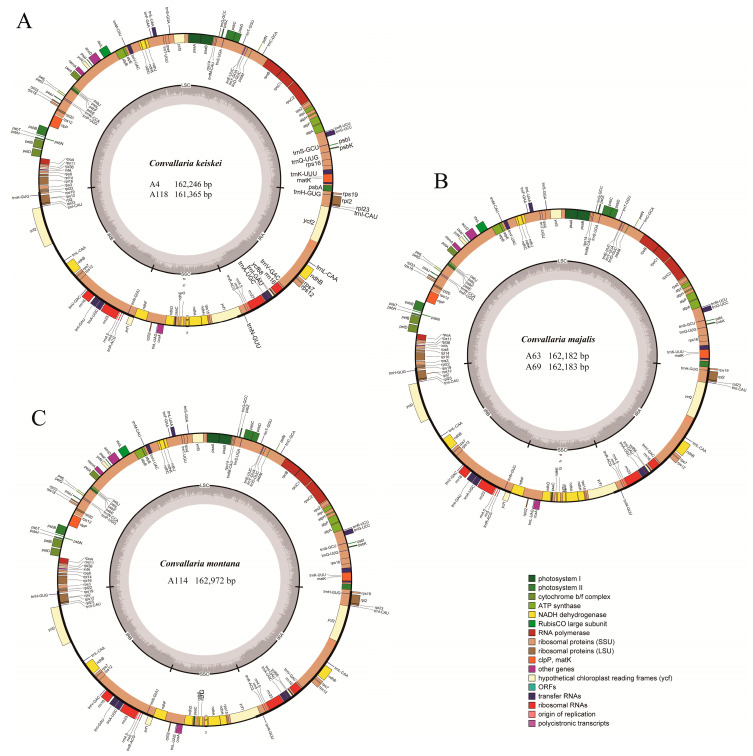
Gene maps of the five *Convallaria* chloroplast genomes. (**A**) Two *C. keiskei* individuals; (**B**) two *C. majalis* individuals; (**C**) one *C. montana* individual. Different color indicates different functional gene groups. The dark gray in the inner represents GC content, and the light gray represents AT content.

Five complete cp genomes of *Convallaria* were assembled with no gaps. The full length of five *Convallaria* chloroplast genomes ranged from 161,365 bp to 162,972 bp (Figure 1, Table 1). All these chloroplast genomes exhibited the typical quadripartite structure, consisting of a pair of IRs (58,280–58,950 bp) separated by the LSC (85,183–85,521 bp) and SSC (17,877–18,502 bp) regions. The total GC content of the five cp genomes were the same (37.9%), whereas the GC content varied in the LSC, SSC, and IR regions as 35.6–35.7%, 31.4–31.6%, and 43.2–43.3%, respectively (Table 1). All cp genomes identically contain 78 protein-coding genes, 30 tRNAs, and four rRNAs. Altogether, the five cp genomes of *Convallaria* were highly conserved in gene content, gene order, and GC content. Nine of the protein-coding genes (*rps16*, *atpF*, *rpoC1*, *petB*, *petD*, *rpl16*, *rpl2*, *ndhB* and *ndhA*) contained one single intron, whereas three genes (*clpP*, *ycf3*, and *rps12*) contained two introns. The *ycf1* gene in IRa was partially duplicated and formed a pseudogene (Table 2).

### 3.2. Variation at IR/SC Boundaries

**Figure 2:**
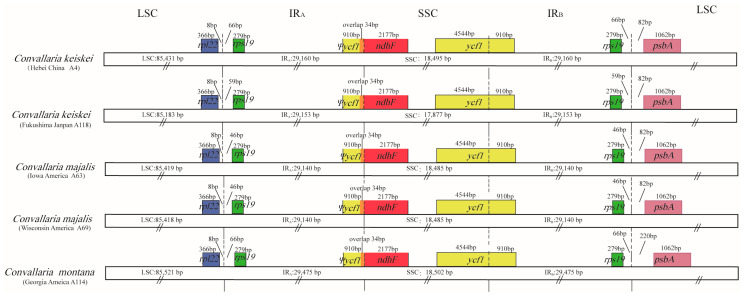
Comparison of LSC, IRs, and SSC junctions among *Convallaria* plastomes.

Comparison of the 5 cp genomes of *Convallaria* revealed minor differences at the IR/LSC boundaries (Figure 2). At the IRA/LSC border, the space length from rpl19 to the border varied from 46 bp to 66 bp. At the IRB/LSC border, the space length from *psbA* to the border was all 82 bp, except in *C. montana* (220 bp). No variation was observed at the IR/LSC boundaries. All the IRb regions expanded 910 bp into *ycf1* and formed a pseudogene Ψ*ycf1* in IRa by duplication. All IRa regions expanded into *ndhF*, causing a 34 bp overlap with *Ψycf1*.

### 3.3. Comparative Analysis of Convallaria cp Genomes and Hotspot Regions Identification

**Figure 3:**
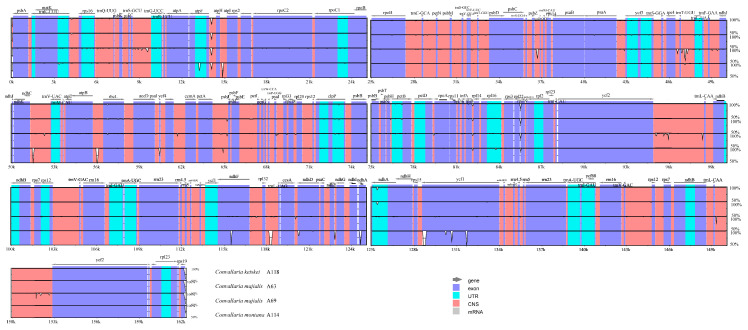
Sequence identity plots among five *Convallaria* chloroplast genomes, with *S. keiskei* as a reference. CNS: conserved non-coding sequences; UTR: untranslated region.

**Figure 4:**
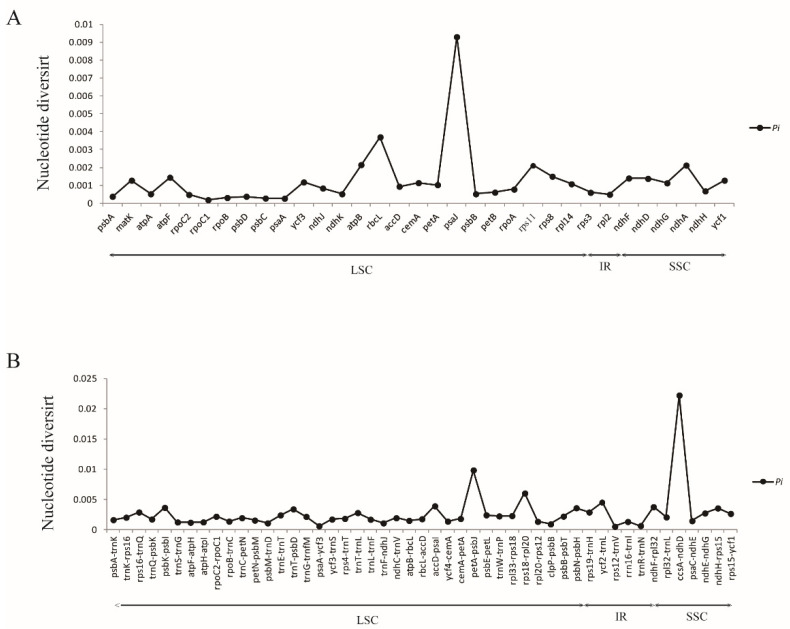
Comparison of nucleotide variability (*Pi*) values in *Convallaria* plastomes. (**A**) *Pi* values among protein-coding genes (CDS). (**B**) *Pi* values among intergenic spacer (IGS) regions.

We analyzed the whole sequence divergence of the five *Convallaria* cp genomes using the mVISTA software with *C. keiskei* (A4) as reference. After alignment, the sequences showed high similarities with only a few regions’ sequence identities less than 90%, suggesting that the cp genomes of *Convallaria* species are conservative (Figure 3). The two IR regions showed a lower level of sequence divergence than LSC and SSC regions. The coding genes and non-coding regions were compared among 5 individuals to detect divergence hotspots. In total, 82 loci (33 coding genes and 49 intergenic spacers) were generated (Figure 4; Table S2), and the nucleotide diversity (*Pi*) value for each locus ranged from 0.00019 (*rpoC1*) to 0.02222 (*ccsA*-*ndhD*). Three regions, *psaJ* (0.0093), *petA*-*psbJ* (0.00985) and *ccsA*-*ndhD* (0.02222), showed a *Pi* > 0.009, which were recognized as hotspot regions that could be applied as molecular markers in species identification and phylogenetic analyses. For all chloroplast genome sequences of Asparagareae, we obtained three hypervariable regions, *petA*-*psbJ* (0.0929), *psbI*-*trnS* (0.08097), and *ccsA*-*ndhD* (0.07816), which could be used in future molecular evolutionary and systematic studies of this family (Table S3).

### 3.4. Long Repeats and Simple Sequence Repeats

**Figure 5:**
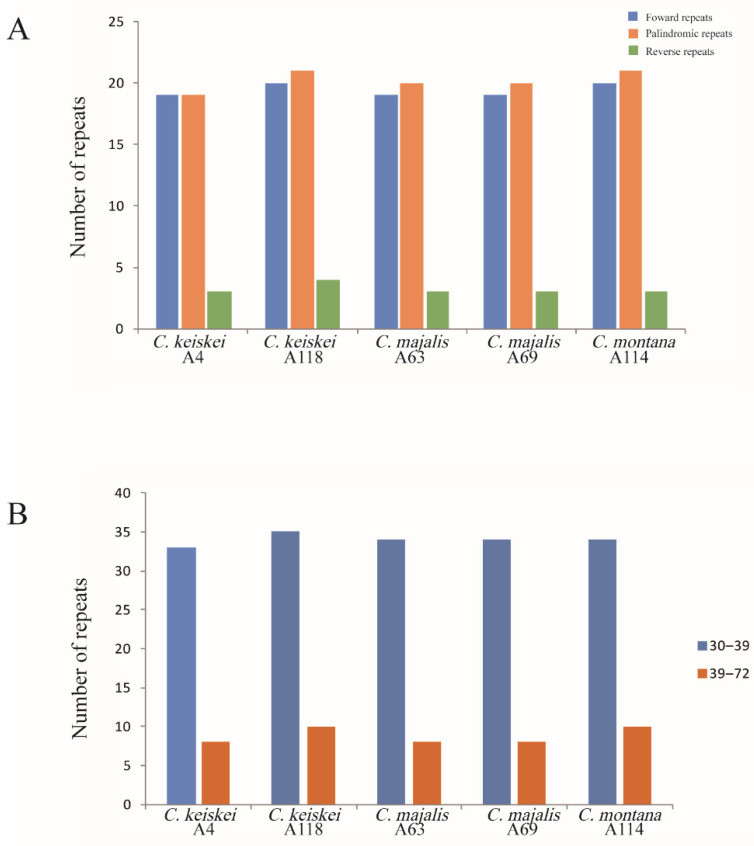
Repeat sequences analysis in five *Convallaria* chloroplast genomes. (**A**) Frequency of repeat types. (**B**) Frequency of repeats by length.

A total of 214 repeats, including 97 forward, 101 palindromic, and 16 reverse repeats, were detected in the five *Convallaria* cp genomes using REPuter (Figure 5A). *Convallaria keiskei* (A118) possessed the most repeats (45), while *C. keiskei* (A4) contained the fewest (41). In all the individuals of *Convallaria*, 79.4% of the repeats ranged from 30 to 39 bp in size (Figure 5B; Table S4). There were 37 repeats shared by the five *Convallaria* cp genomes. Additionally, 3 and 4 repeats were unique in *C. keiskei* (A118) and *C. montana* (A114), respectively (Table S5).

**Figure 6:**
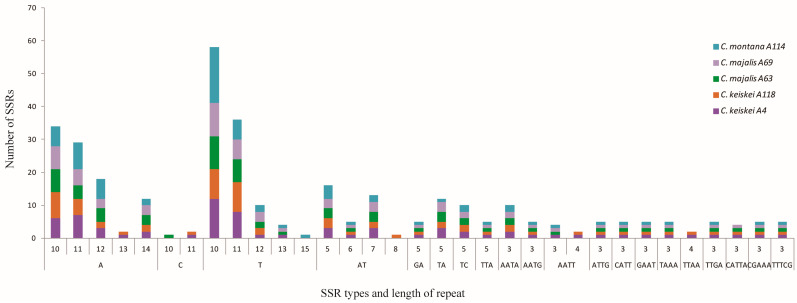
Distribution of simple sequence repeats (SSRs) in the five *Convallaria* chloroplast genomes.

A total of 338 SSRs were identified by MISA analysis across the five *Convallaria* cp genomes. The number of SSRs ranged from 64 (*C. majalis* A69) to 74 (*C. montana* A114) SSRs, with 21 SSRs shared among all genomes (Table S6). Among these SSRs, more than one-half (60.35%) were composed of A/T bases (Figure 6). In general, the SSRs of these cp genomes showed abundant variation, which can be used in population genetics study of these *Convallaria* species.

### 3.5. Phylogenetic Analysis

**Figure 7:**
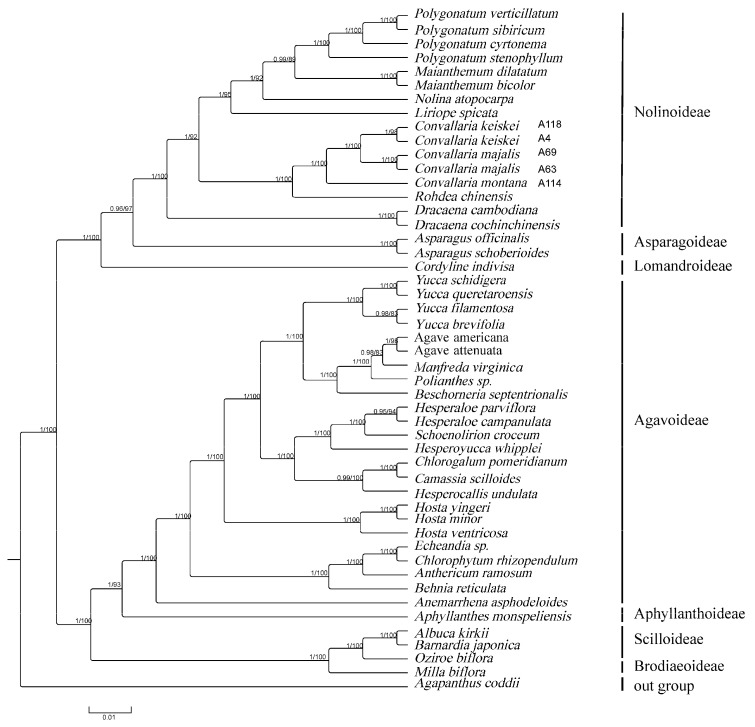
Phylogenetic relationships of Asparagaceae reconstructed using Bayesian inference (BI) and maximum likelihood (ML) based on 68 common CDS. Support values are indicated above the branches. PP, posterior probability; BS, bootstrap support.

We used 48 cp genome sequences of Asparagareae and 1 outgroup of Agapanthoideae in total for phylogenetic analysis. The trees reconstructed with the 68 common CDSs shared between the plastomes displayed almost identical topologies with generally high support values, in both ML and BI analyses. The phylogenetic tree was almost fully supported (PP/BS = 1/100) at all nodes (Figure 7). The phylogenetic tree indicated that the subfamilies Scilloideae and Brodiaeoideae constituted the earliest diverging lineage in Asparagaceae. All these phylogenetic trees identically supported the monophyly of *Convallaria*, which in turn formed a sister clade to the *Rohdea*. Within *Convallaria*, *C. keiskei* was resolved as a monophyletic clade with a sister relationship to *C. majalis*, and *C. montana* was at the basal position.

## 4. Discussion

Comparative analysis results indicate that five cp genome sequences of *Convallaria* showed highly conserved genomic structures. No variation and rearrangement of the gene content were found between the five cp genomes of *Convallaria*. All plastomes had the same number of protein-coding genes, tRNAs and rRNAs. Genes of *ycf1* and *infA* were found to be pseudogenes in five individuals. The pseudogenizations of *ycf1* and locations of ψ*ycf1* copies commonly found in other plants [43,44]. The pseudogene was firstly thought to have lost the ability of protein coding [45] but was now considered as an evolutionary relic of the functional gene [46]. Such conservatism revealed accords with the low substitution rate of chloroplast genomes and the presumed recent divergence within genus *Convallaria*. Similar findings were also found in other closely related species [47,48]. Within cp genomes, the results of comparative analysis indicated that CDS and IRs regions were more conserved than IGS and SCs, correspondingly. The IR regions are highly conserved, which is important for the stabilization of the chloroplast genome structure [49]. Comparison of the IR/SC boundary areas among species suggested expansions and contractions of the IR region. The expansion and contraction of the IR regions often results in the length change of cp genomes [50]. The mechanism of larger IRs expansion may be caused by the double-strand break repair (DSBR) [51]. Because of the conservatism, the IR regions showed a lower level of sequence divergence than LSC and SSC regions in *Convallaria* cp genomes, in accordance with other studies [52,53,54].

The polymorphic cp DNA non-coding regions have been widely used to investigate species identification and molecular phylogeny at the interspecific level [55,56]. We have detected three variable regions (*petA*-*psbJ*, *psbI*-*trnS* and *ccsA*-*ndhD*) that can be used in species identification and phylogeny. SSR is a repetitive sequence consisting of simple repeating units in tandem and has been widely used as molecular marker in genetic structure and genetic diversity analysis [57,58]. The SSRs detected in present study showed abundant variation, and can therefore be applied in the genetic diversity, population genetics analyses [59,60].

In recent years, chloroplast genomes have been widely used to evaluate the relationship of closely related species in taxonomic studies. For example, cp genomes of 35 species representing 31 genera from Ranunculaceae were sequenced and utilized to clarify the long-standing systematic controversies of this family [22]. Our phylogenetic analyses based on 48 cp genomes successfully resolved intergeneric relationships within Asparagaceae. We obtained a well-resolved and robust phylogenetic tree. Two main clades including seven subfamilies were confirmed: Lomandroideae + (Asparagoideae + Nolinoideae) and Brodiaeoideae + (Scilloideae + (Aphyllanthoideae + Agavoideae)). The first two diverged clades were Lomandroideae and Brodiaeoideae, respectively. This result was congruent with previous research [18,61]. *Convallaria* was confirmed as a monophyly most closely related to the genus *Rohdea*. The *Convallaria* clade consisted of two lineages: one contains the North American species *C. montana*, and another contains two Eurasian distributed species, *C. keiskei* and *C. majalis*. A similar phylogenetic relationship was revealed in other plant taxa that displayed an Eastern Asian–Eastern North American disjunct distribution, such as *Croomia*, *Polygonatum*, *Maianthemum* [62,63,64]. This prevalent phylogeographic pattern in plants was explained as the result of vicariance events after dispersal via the Bering or North Atlantic land bridge during the Late Tertiary [65,66,67,68].

## 5. Conclusions

In conclusion, our findings reveal the detailed characteristics of the complete cp genome of three *Convallaria* species. The gene content, gene order, and gene orientation are highly conserved. Comparative analyses revealed that no rearrangements occurred in *Convallaria,* and that intergenic regions were more variable than coding regions. Three hypervariable regions (*petA*-*psbJ*, *psbI*-*trnS* and *ccsA*-*ndhD*) were identified as valuable molecular markers. The cp genome data provided strong support for the relationships within *Convallaria* and among the subfamily clades within Asparagareae, which proved the cp genome to be useful genetic resources in dealing with phylogenetically difficult taxa. The complete cp genome sequences will contribute to further studies in molecular identification, genetic diversity, and phylogeny.

## Figures and Tables

**Table 1:** The sample information and characteristics of *Convallaria* chloroplast genomes.

Characteristics	*C. keiskei* (A4)	*C. keiskei*(A118)	*C. montana* (A114)	*C. majalis* (A63)	*C. majalis* (A69)
Location	China: Hebei	Japan: Fukushima	USA: Georgia	USA: Iowa	USA: Washington
Latitude (N)	30°12′1″	35°43′6″	29°43′55″	30°44′27″	28°48′21″
Longitude (E)	120°71′6″	139°44′47″	121°5′10″	116°27′9″	120°54′47″
Total cp DNA Size (bp)	162,246	161,365	162,972	162,183	162,182
LSC length (bp)	85,432	85,183	85,521	85,419	85,418
SSC length (bp)	18,495	17,877	18,502	18,485	18,485
IR length (bp)	29,160	29,153	29,475	29,140	29,140
Total GC content (%)	37.9	37.9	37.9	37.9	37.9
LSC	35.6	35.7	35.7	35.6	35.6
SSC	31.4	31.6	31.4	31.4	31.4
IR	43.2	43.2	43.3	43.2	43.2
Total number of genes	133	133	133	133	133
Protein-coding genes	85	85	85	85	85
rRNAs genes	8	8	8	8	8
tRNAs genes	38	38	38	38	38
Duplicated genes	20	20	20	20	20

IR, inverted repeat region; LSC, large single-copy region; SSC, small single-copy region.

**Table 2:** Gene list of the *Convallaria* chloroplast genomes.

Groups of Gene	Name of Genes
Ribosomal RNAs	*rrn16*(×2)*, rrn23*(×2)*, rrn4.5*(×2)*, rrn5*(×2)
Transfer RNAs	*trnA-UGC*(×2)*, trnC-GCA, trnD-GUC, trnE-UUC, trnF-GAA, trnfM-CAU, trnG-GCC, trnG-UCC, trnH-CAU, trnH-GUG, trnI-CAU*(×2)*, trnI-GAU*(×2)*, trnK-UUU, trnL-CAA*(×2)*, trnL-UAA, trnL-UAG, trnM-CAU, trnN-GUU*(×2)*, trnP-UGG, trnQ-UUG, trnR-ACG*(×2)*, trnR-UCU, trnS-GCU, trnS-GGA, trnS-UGA, trnT-GGU, trnT-UGU, trnV-GAC*(×2)*, trnV-UAC, trnW-CCA, trnY-GUA*
Photosystem I	*psaA*, *psaB*, *psaC*, *psaI*, *psaJ*
Photosystem II	*psbA*, *psbB*, *psbC*, *psbD, psbE, psbF, psbH, psbI, psbJ, psbK, psbL, psbM, psbN, psbT*
Cytochrome	*psbA*, *psbB*, *psbC*, *psbD, psbE, psbF, psbH, psbI, psbJ, psbK, psbL, psbM, psbN, psbT*
ATP synthase	*atpA, atpF ^a^, atpH, atpI, atpE, atpB*
Rubisco	*bri* *ck*
NADH dehydrogenase	*ndhJ, ndhK, ndhC, ndhB ^a^* (×2)*, n* *dhF, ndhD, ndhE, ndhG, ndhI, ndhA ^a^, ndhH*
ATP-dependent protease subunit P	*clpP ^b^*
Chloroplast translational initiation factor	*i* *nfA*
Chloroplast envelope membrane protein	*cemA*
Large units	*rpl33, rpl20, rpl36, rpl14, rpl16 ^a^, rpl2, rpl2 ^a^* (×2)*, rpl23*(×2)*, rpl32*
Small units	*rps16 ^a^, rps2, rps14, rps4, rps18, rps12 ^b^* (×2)*, rps11, rps8, rps19, rps3, rps7* (×2)*, rps15*
RNA polymerase	*rpoC2, rpoC1 ^a^, rpoB, rpoA*
Miscellaneous proteins	*matK, aacD, ccsA*
Hypothetical proteins and conserved reading frame	*ycf1, ycf2*(×2)*, ycf3 ^b^, ycf4*
Pseudogenes	*Ψy* *cf* *1, Ψ* *infA*

^a^ Indicates the genes containing one single intron. ^b^ Indicates the genes containing two introns. (×2) indicates genes duplicated in the IR regions. Ψ indicates pseudogene.

## Data Availability

The data which support the findings of this study are openly available in GenBank of NCBI at https://www.ncbi.nlm.nih.gov, accessed on 2 June 2022, reference number (*C. keiskei* A4, ON645923; *C. keiskei* A118, ON303655; *C. majalis* A63, ON303653; *C. majalis* A69, ON645922 and *C. montana* A114, ON303654).

## Supplementary Materials

The following supporting information can be downloaded at: https://www.mdpi.com/article/10.3390/genes13101724/s1; Table S1: Accession numbers of chloroplast genomes used for phylogenetic analyses; Table S2: Nucleotide variability (*Pi*) values and total number of mutation (*Eta*) in *Convallaria*; Table S3: Nucleotide variability (*Pi*) values and total number of mutation (*Eta*) in Asparagareae; Table S4: Repeat types and lengths of *Convallaria* chloroplast genomes; Table S5: Analyses of repeat sequences in five *Convallaria* chloroplast genomes; Table S6: Simple sequence repeat (SSR) polymorphism in five *Convallaria* chloroplast genomes.
