# Supplementary material for: Evolutionary Comparison of the Complete Chloroplast Genomes in Convallaria Species and Phylogenetic Study of Asparagaceae
Source: Genes (Basel). 2022 Sep 26;13(10):1724. doi: 10.3390/genes13101724 (PMC9601677; doi:10.3390/genes13101724)
Supplement: Supplementary file 1 [file genes-13-01724-s001.zip › Table S1.pdf]

Table S1. Accession numbers of chloroplast genomes used for phylogenetic analyses

Family	Subfamily	Species	Genbank accession numbers
Asparagaceae	Nolinoideae	*Polygonatum verticillatum*	KT722981
Asparagaceae	Nolinoideae	*Polygonatum sibiricu*	KT695605
Asparagaceae	Nolinoideae	*Polygonatum cyrtonema*	KT630835
Asparagaceae	Nolinoideae	*Polygonatum stenophyllum*	KX822773
Asparagaceae	Nolinoideae	*Nolina atopocarpa*	KX931462
Asparagaceae	Nolinoideae	*Maianthemum dilatatum*	MF150041
Asparagaceae	Nolinoideae	*Maianthemum bicolor*	KX790362
Asparagaceae	Nolinoideae	*Liriope spicata*	MH680945
Asparagaceae	Nolinoideae	*Rohdea chinensis*	MH356725
Asparagaceae	Nolinoideae	*Dracaena cambodiana*	MH293451
Asparagaceae	Nolinoideae	*Dracaena cochinchinensis*	MF943127
Asparagaceae	Asparagoideae	*Asparagus officinalis*	KY364194
Asparagaceae	Asparagoideae	*Asparagus schoberioides*	KX790361
Asparagaceae	Lomandroideae	*Cordyline indivisa*	KX822776
Asparagaceae	Agavoideae	*Yucca schidigera*	KX931469
Asparagaceae	Agavoideae	*Yucca queretaroensis*	KX931468
Asparagaceae	Agavoideae	*Yucca filamentosa*	KX931467
Asparagaceae	Agavoideae	*Yucca brevifolia*	KX931466
Asparagaceae	Agavoideae	*Polianthes sp.*	KX931464
Asparagaceae	Agavoideae	*Agave americana*	KX519714
Asparagaceae	Agavoideae	*Manfreda virginica*	KX931461
Asparagaceae	Agavoideae	*Agave attenuata*	KX931447
Asparagaceae	Agavoideae	*Beschorneria septentrionalis*	KX931451
Asparagaceae	Agavoideae	*Hesperaloe parviflora*	KX931457
Asparagaceae	Agavoideae	*Hesperaloe campanulata*	KX931456
Asparagaceae	Agavoideae	*Schoenolirion croceum*	KX931465
Asparagaceae	Agavoideae	*Hesperoyucca whipplei*	KX931459
Asparagaceae	Agavoideae	*Chlorogalum pomeridianum*	KX931453
Asparagaceae	Agavoideae	*Camassia scilloides*	KX931452
Asparagaceae	Agavoideae	*Hesperocallis undulata*	KX931458
Asparagaceae	Agavoideae	*Hosta yingeri*	MF990205
Asparagaceae	Agavoideae	*Hosta minor*	KX822777
Asparagaceae	Agavoideae	*Hosta ventricosa*	KX931460
Asparagaceae	Agavoideae	*Echeandia sp.*	KX931455
Asparagaceae	Agavoideae	*Chlorophytum rhizopendulum*	KX93454
Asparagaceae	Agavoideae	*Anthericum ramosum*	KX790364
Asparagaceae	Agavoideae	*Behnia reticulata*	KX931450
Asparagaceae	Agavoideae	*Anemarrhena asphodeloides*	KX931449
Asparagaceae	Aphyllanthoideae	*Aphyllanthes monspeliensis*	KX790360
Asparagaceae	Scilloideae	*Albuca kirkii*	KX931448

Asparagaceae	Scilloideae	*Barnardia japonica*	KX822775
Asparagaceae	Scilloideae	*Oziroe biflora*	KX931463
Asparagaceae	Brodiaeoideae	*Milla biflora*	KX822778
Asparagaceae	Agapanthoideae	*Agapanthus coddii*	KX790363
