# Supplementary material for: Evolutionary Comparison of the Complete Chloroplast Genomes in Convallaria Species and Phylogenetic Study of Asparagaceae
Source: Genes (Basel). 2022 Sep 26;13(10):1724. doi: 10.3390/genes13101724 (PMC9601677; doi:10.3390/genes13101724)
Supplement: Supplementary file 1 [file genes-13-01724-s001.zip › Table S2.pdf]

Table S2: Nucleotide variability (*Pi*) values and total number of mutation (*Eta*) in *Convallaria*.

Name	Length	Eta	Pi
psbA-trnK	244	1	0.00164
trnK-rps16	692	3	0.00204
rps16-trnQ	1314	8	0.0029
trnQ-psbK	345	1	0.00174
psbK-psbI	439	4	0.00366
trnS-trnG	1318	4	0.00125
atpF-atpH	523	1	0.00121
atpH-atpI	526	1	0.00126
rpoC2-rpoC1	182	1	0.0022
rpoB-trnC	1157	3	0.00139
trnC-petN	923	3	0.00196
petN-psbM	897	3	0.00156
psbM-trnD	726	2	0.0011
trnE-trnT	498	3	0.00242
trnT-psbD	767	5	0.00341
trnG-trnfM	185	1	0.00217
psaA-ycf3	637	1	0.00063
ycf3-trnS	578	2	0.00173
rps4-trnT	344	1	0.00184
trnT-trnL	1018	6	0.00281
trnL-trnF	353	1	0.0017
trnF-ndhJ	740	2	0.0011
ndhC-trnV	1501	6	0.00195
atpB-rbcL	765	2	0.00152
rbcL-accD	669	2	0.00179
accD-psaI	306	3	0.00392
ycf4-cemA	869	3	0.00139
cemA-petA	218	1	0.00183
petA-psbJ	1102	23	0.00985
psbE-petL	1316	6	0.00243
trnW-trnP	178	1	0.00225
rpl33-rps18	177	1	0.00231
rps18-rpl20	269	4	0.00604
rpl20-rps12	743	2	0.00135
clpP-psbB	428	1	0.00093
psbB-psbT	181	1	0.00222
psbN-psbH	111	1	0.0036
rps19-trnH	138	1	0.0029
ycf2-trnL	3668	43	0.00478
rps12-trnV	1870	2	0.00054
rrn16-trnI	301	1	0.00133

trnR-trnN	609	1	0.00066
ndhF-rpl32	957	8	0.00378
rpl32-trnL	937	4	0.00209
ccsA-ndhD	207	11	0.02222
psaC-ndhE	410	1	0.00147
ndhE-ndhG	178	1	0.00274
ndhH-rps15	112	1	0.00357
rps15-ycf1	301	2	0.00266
