# Supplementary material for: Evolutionary Comparison of the Complete Chloroplast Genomes in Convallaria Species and Phylogenetic Study of Asparagaceae
Source: Genes (Basel). 2022 Sep 26;13(10):1724. doi: 10.3390/genes13101724 (PMC9601677; doi:10.3390/genes13101724)
Supplement: Supplementary file 1 [file genes-13-01724-s001.zip › Table S3.pdf]

Table S3: Nucleotide variability (*Pi*) values and total number of mutation (*Eta*) in Asparagareae.

Name	Length	Eta	Pi
petA-psbJ	1388	126	0.0929
psbI-trnS	158	36	0.08097
ccsA-ndhD	196	47	0.07816
ndhE-ndhG	226	45	0.06919
trnK-rps16	916	88	0.0678
atpF-atpH	765	115	0.06765
trnC-petN	1241	59	0.06439
ycf3-trnS	747	58	0.06277
psbM-trnD	1595	142	0.06217
psaJ-rpl33	708	60	0.06213
ndhG-ndhI	483	56	0.06127
ycf4-cemA	682	84	0.06097
psbK-psbI	447	24	0.06088
petN-psbM	1190	11	0.05903
trnW-trnP	217	53	0.0583
psaC-ndhE	1011	94	0.05786
trnF-ndhJ	1086	118	0.05785
rps18-rpl20	291	37	0.05519
ndhH-rps15	176	31	0.05501
psaI-ycf4	523	132	0.05478
rps15	279	89	0.05437
matK	1575	549	0.05267
trnT-psbD	1670	182	0.05172
trnE-trnT	1362	104	0.04976
rpoB-trnC	1562	116	0.04844
atpB-rbcL	829	89	0.04841
psbA-trnK	320	61	0.0484
trnfM-rps14	204	35	0.04814
rpl22	435	107	0.04805
psbC-trnS	190	30	0.04739
atpI-rps2	436	75	0.04641
rps8-rpl14	439	57	0.04605
ndhF	2453	595	0.04528
rbcL	1597	677	0.04524
trnG-trnfM	237	20	0.04462
cemA-petA	253	70	0.04426
petB-petD	276	59	0.04393
rps16-trnQ	1839	70	0.04388
psbZ-trnG	454	69	0.04372
ndhF-rpl32	1323	19	0.04329
trnP-psaJ	428	57	0.04322

petL-petG	220	60	0.04243
clpP	2819	556	0.04064
ccsA	978	256	0.04055
ndhA	2482	501	0.04052
accD-psaI	1059	56	0.04
atpH-atpI	1053	74	0.03871
ycf2-trnL	3988	129	0.0384
psbE-petL	1639	191	0.03829
clpP-psbB	795	115	0.03828
ndhG	534	141	0.03823
rpl33-rps18	268	35	0.03782
rpl20-rps12	894	150	0.03769
psbB-psbT	571	46	0.03744
atpB	1496	449	0.03665
ndhH	1182	234	0.0362
rps4-trnT	439	84	0.03598
ndhD	1541	371	0.03586
rpl32-trnL	1243	7	0.03375
rpl20	367	76	0.03364
trnQ-psbK	363	99	0.03347
rps11	424	100	0.03274
atpF	1671	295	0.03198
ndhE	306	58	0.03194
psbK	196	35	0.03187
rps3	687	160	0.03176
rps14-psaB	161	32	0.0316
ndhI	543	104	0.03137
rpoC2	4192	935	0.03099
petG-trnW	149	22	0.03089
petB	1576	271	0.02987
psaA-ycf3	289	55	0.02955
cemA	705	160	0.0292
rpl32	191	39	0.02899
accD	2061	282	0.02886
atpA	1524	277	0.02779
rpl33	210	32	0.02704
rpoA	1033	223	0.02676
petD	1455	199	0.02647
trnS-rps4	406	18	0.02626
trnS-psbZ	434	34	0.02588
rpoC1	2906	499	0.02534
ycf3	2152	351	0.02523
rpl36	114	18	0.02511
rps2	715	132	0.02503

ndhK	888	148	0.02474
ycf4	555	79	0.02355
rpl14	369	59	0.02321
psbN	133	18	0.0229
rpoB	3225	506	0.02263
psbB	1527	219	0.02226
ndhJ	477	82	0.02197
petA	969	143	0.02154
petL	96	12	0.02117
rps4	608	90	0.02071
ndhC	368	51	0.02052
psaB	2205	247	0.02008
rps18	408	53	0.02002
rps8	405	79	0.02
psbC	1428	179	0.01977
psbH	223	36	0.01962
rps14	308	46	0.01861
psaA	2253	277	0.01853
psaJ	131	14	0.01824
atpI	744	82	0.01781
psbA	1062	140	0.01758
psbD	1062	115	0.01668
psbM	108	10	0.01663
psbJ	123	12	0.01524
psaC	246	34	0.01491
petG	114	12	0.01409
psbI	121	13	0.01324
psbT	103	5	0.01324
trnL-ndhB	648	45	0.01169
ycf2	7277	557	0.0116
psbZ	189	14	0.01078
psaI	111	6	0.01
atpH	249	22	0.00974
trnR-trnN	674	39	0.00929
petN	90	8	0.00915
psbL	117	7	0.00893
psbE	252	22	0.00744
ndhB-rps7	351	16	0.00668
rps7	468	36	0.00649
rpl2	1502	80	0.0064
psbF	120	9	0.00544
rpl23	318	11	0.00486
ndhB	2237	90	0.00463
