# Supplementary material for: Evolutionary Comparison of the Complete Chloroplast Genomes in Convallaria Species and Phylogenetic Study of Asparagaceae
Source: Genes (Basel). 2022 Sep 26;13(10):1724. doi: 10.3390/genes13101724 (PMC9601677; doi:10.3390/genes13101724)
Supplement: Supplementary file 1 [file genes-13-01724-s001.zip › Table S4.pdf]

Table S4: Repeat types and lengths of *Convallaria* chloroplast genomes.

	C. keiskei A4	*C. keiskei* A118	*C. majalis* A63	*C. majalis* A69	*C. montana* A114
Foward repeats	19	20	19	19	20
Palindromic repeats	19	21	20	20	21
Reverse repeats	3	4	3	3	3
Complimentary repeats	0	0	0	0	0
30-39 bp	33	35	34	34	34
40-72 bp	8	10	8	8	10
